# Human rights and reproductive health: political realities and pragmatic choices for married adolescent women living in urban slums, Bangladesh

**DOI:** 10.1186/1472-698X-11-S3-S3

**Published:** 2011-12-16

**Authors:** Sabina Faiz Rashid

**Affiliations:** 1 James P Grant School of Public Health, BRAC University, Dhaka 1212, Bangladesh

## Abstract

**Background:**

In Bangladesh, particularly in urban slums, married adolescent women’s human rights to life, health, and reproductive and sexual health remain adversely affected because of the structural inequalities and political economic, social and cultural conditions which shape how rights are understood, negotiated and lived.

**Methods:**

The focus of the research and methods was anthropological. An initial survey of 153 married adolescent women was carried out and from this group, 50 in-depth interviews were conducted with selected participants and, from the in-depth interviews, a further eight case studies of women and their families were selected for in-depth repeated interviews and case histories.

**Results:**

This paper speaks of the unanticipated complexities when writing on reproductive rights for poor adolescent women living in the slums, where the discourses on ‘universal human rights’ are often removed from the reality of adolescent women’s everyday lives. Married adolescent women and their families remain extremely vulnerable in the unpredictable, crime-prone and insecure urban slum landscape because of their age, gender and poverty. Adolescent women’s understanding of their rights such as the decision to marry early, have children, terminate pregnancies and engage in risky sexual behaviour, are different from the widely accepted discourse on rights globally, which assumes a particular kind of individual thinking and discourse on rights and a certain autonomy women have over their bodies and their lives. This does not necessarily exist in urban slum populations.

**Conclusions:**

The lived experiences and decisions made pertaining to sexual and reproductive health and ‘rights’ exercised by married adolescent women, their families and slum communities, allow us to reflect on the disconnect between the international legal human rights frameworks as applied to sexual and reproductive health rights, and how these are played out on the ground. These notions are far more complex in environments where married adolescent women and their families live in conditions of poverty and socioeconomic deprivation.

## Background

In the 1990s, women’s reproductive rights were recognized globally and nationally, with a focus on political, economic, social, and cultural rights at both individual and collective levels at the International Conference on Population Development (ICPD) in 1994 [1]. ICPD outlined the right to reproductive autonomy, including the right to privacy and the right to decide the number and spacing of one’s children, which ideally should obligate governments that are signatories to ensure that men and women have equal access to a full range of contraceptive choices and reproductive health services; they have access to information and that their decisions pertaining to their reproductive choices are fully respected by the government and third parties [2].

If one were to look at how the concept of reproductive rights has been translated into policies and programmes in the years since the development of the ICPD agenda, Bangladesh like its neighbouring country of India, adopted the ICPD definition of reproductive health, and as signatories initiated policy reforms to reflect this new focus, but less was accomplished in implementing integrated reproductive health programmes on the ground. Several challenges remained, such as improving knowledge and support among stakeholders, planning for integration and decentralised services, developing human resources, improving quality of care and maintaining the ICPD Cairo agenda which was to create an enabling environment for the implementation of sexual and reproductive health and rights [3]. Although the Bangladesh government signed up to ICPD, it struggled to deliver. There were problems with creating a more enabling climate for a rights based approach. Moreover, since ICPD, global funding for sexual and reproductive health (SRH) programmes has fallen short of the financial targets agreed. In 2004, a gap of over US $3 billion was estimated with serious adverse SRH consequences for the millions of women on the ground [4].

In Bangladesh, particularly in urban slums, women’s human rights to life, health, and reproductive health remain adversely affected because of the structural inequalities and social and cultural conditions which shape how rights are understood, negotiated and lived. In this paper, I show how reproductive rights and well-being cannot be addressed without first addressing the context of extreme poverty, hunger and violence threatening men’s and women’s survival.

Given the unaffordable housing and rising land prices, Dhaka city is filled with large urban slums, housing the poorest migrants and residents. These slums are characterised by poor law and order, lack of access to basic services such as water, sanitation and drainage, and located in flood-prone areas. As most slums are not recognized legally, the government, non-government organizations (NGOs) and donors generally do not provide services in these areas. A survey of 9,048 slum settlements in six cities in Bangladesh found that more than half did not have access to fixed garbage disposal and had no mechanism for garbage collection. 70% of slum settlements had no access to safe latrines, and in half of the cases, the latrines were shared by at least six families (30 or more persons) [5]. Another study reported that only 43 of 1,925 slum settlements identified were within 100 metres of a public toilet. The largest slum settlement with more than 12,000 households did not have a single public toilet or health clinic [6]. Despite the growth of urban slums, the country does not have a comprehensive policy on urbanization and urban poverty [5,6]. Living in slum settlements means poor residents are exposed to organised crime, drugs and gang violence at an early age [7]. Patronage relationships characterise slum politics, extending from the slum all the way into the local authority and political parties. A World Bank report on crime and violence in four slums in Dhaka found that 93% slum residents reported they had been affected by crime and violence over the last 12 months (prior to the survey) with 33 different types of crime. Among the most commonly reported crimes are enforced toll collection, local thug, or mafia-style induced violence, illegal drugs and alcohol and arms businesses, land grabbing, gambling, domestic violence against women and children, arson, murder and kidnapping [6].

This paper examines the complexities of writing on reproductive rights for poor adolescent women living in the slums, where the discourses on ‘universal human rights’ are often removed from the reality of young women’s everyday lives. Many young women live in shack settlements built on vacant or disused government or private land, never knowing when the settlements might be forcibly removed. Married adolescent women living in this environment are extremely vulnerable in this unpredictable, crime-prone and insecure landscape because of their age, gender and poverty. Their lived experiences and decisions pertaining to marriage, relationships, bearing children and pregnancy terminations, allow us to reflect on the disconnect between the international legal human rights frameworks as applied to sexual and reproductive health and rights (SRHR), and how these are played out on the ground. Gendered realities and structural constraints mean that universal perspectives on rights are not necessarily the starting point for many young women.

## Methods

The study was undertaken in three sections of a large urban slum, Phulbari. It was located in Pollabi, formerly part of the Mirpur area of Dhaka. Mirpur presents a classic case of income inequality, with predominantly middle-class people living in this area, on one hand, and very poor populations living in squatter settlements, on the other. It has the highest proportion of squatter households in Dhaka city, with most of the poor resettled here after being forcibly evicted in 1975 from different parts of the city [8]. Fieldwork was carried out from December 2001 to January 2003. However, the entire slum was evicted on July 25, 2002 and I continued my fieldwork with many of the young women and their families in their new places of residence. Some moved to nearby areas and some set up temporary settlements in the evicted area. I undertook case studies, in-depth interviews and long-term participant observation, providing rich empirical data. In addition, a survey was carried out to gather general background information, including young women’s reproductive histories. My fieldwork was limited to certain sections of the slums [1,2,4] and I enlisted the assistance of health providers (who lived and worked in the slum) to identify and locate married adolescent women. As members of the community, they provided legitimacy and instant access, which might have taken months to build. Their presence in the initial door to door visits meant that community members, including young women, were much more willing to speak to my research assistant and I. The health workers introduced us to the main ‘gatekeepers’ of the slum – prominent leaders of the community who gave permission to work there. The health workers themselves were the second set of gatekeepers to access married adolescent women and eventually they became key informants. To gain a general understanding of the women’s situation, an initial survey of 153 young women was carried out. The survey covered socio-demographic data, reproductive histories and young women’s experiences of health and reproductive illnesses. Women were initially selected for the survey, taking a starting point (e.g. for section 1 we relied on the location of the mosque inside this slum as a beginning point) and then randomly selected every third household. However, in reality, we had to skip a house and move to the next one if women were unwilling to speak to us.

The focus of my research and methods was anthropological. I conducted 50 in-depth interviews selected from the survey participants and, from the in-depth interviews, selected a further eight case studies of women and their families. These included discussions with their husbands. Selection was based on richness of the data collected in the interviews, the willingness of young women to talk further and their families’ availability, and the existing rapport with their family members, including spouses, in-laws and neighbors. All of these people were at times the gatekeepers to accessing the adolescent women. The aim of the surveys, in-depth interviews, case studies and observations was to obtain an in-depth understanding of the lives of adolescent women and their families.

After the eviction took place, I managed to follow up several of the young women and their families in their new homes. This allowed me to understand some of the social and economic impacts of eviction, including the breakdown of social networks and relationships. Given the conditions and environment of the slum, I relied on information volunteered without pressing or probing, going from door to door in the three sections of Phulbari slum. The research process was not linear but an on-going process and I often moved back and forth between repeated in-depth interviews and conversations, case studies and fieldwork observations. Many of the married adolescent women and community members shared personal and intimate stories and events. Through the use of multiple repeated interviews, observations and conversations, I pieced together much of the social and political-economic conditions of slum life as it impacts on young women’s lives and shapes their reproductive behaviour. The data were continually reviewed and checklists and questions were changed to adapt to issues raised by the adolescent women and their families. Prolonged engagement resulted in rich data sets and personal relationships were formed. It is argued that if a researcher is in a site for some time, as a “living, reacting fellow human being, rather than a human pretending to be a disembodied fly on the wall, the people you are studying will create a space, a role for you.” [9] [p180]. I reacted to what went on in the slum and in the lives of the married adolescent women. I agreed with some opinions and statements and disagreed with others. I showed approval or disapproval of some behaviours. I even intervened in household fights and disputes among neighbours [9]. Over time, my status gradually changed from being a complete outsider to being close to an adopted family member, a privileged but sympathetic outsider [10]. The research received ethical clearance from The Australian National University (Australia) and World Health Organisation, Geneva (funding agency) and all ethical processes of confidentiality and informed consent were followed. All names used in the paper are pseudonyms.

## Results

### Mastaans, drugs and gang violence: impact on adolescent women’s lives

In Phulbari slum settlements, a climate of violence existed in all the sections, leaving residents in a constant state of tension and fear. During the first six months of fieldwork, there were two police raids, a bloody gang war among two rival factions, bomb threats, a kidnapping of a young woman, and the murder of a young boy. In addition, drug addicts roamed certain parts of the slums, and loud arguments and violent fights were common among landlords and tenants, neighbours, family members, and husbands and wives. It was not unusual during fieldwork to find all the local shops shut and the place looking deserted after a police raid; or an eerie quietness prevailing in some of the seedier and noisy areas, after a bomb threat or violence stemming from a drug deal turning sour.

Virtually every slum had its own informal leadership structure, with self appointed political leaders (referred to as mastaans) – thugs, who have strong links to influential government politicians, police and local government authorities, a finding supported by other studies [7,11]. The relationships were fluid with alliances changing depending on the needs of leaders and other individuals and prevailing politics within the slum. The self-appointed leaders belonged to different political parties, and the political parties relied in turn on this local leadership for electoral support and re-election of particular candidates in an area. A leader or mastaan’s power base was further consolidated if the political party he belonged to formed the government. Bangladesh has two main political parties, the Bangladesh National Party [BNP] and Awami League (AL), run by two women leaders, which have dominated national politics for more than two decades now. Their confrontational politics and rivalry have affected the political situation. As noted, patronage relationships characterise slum politics and extend from the slum all the way into the local authority and political parties [11].

Gang enmity and feuding were common in the slums, and seriously affected the lives of adolescent women and their families. In the 50 in-depth interviews, a number of adolescent women reported that their husbands were or had been gang members. One of them was murdered in 2001 leaving his wife Shilpi widowed at the age of 16 and petrified of leaving her home. Another young man was left disabled after a gang fight, and his 17 year old wife, Lucky, was forced to work and rely on the support of gang members, thereby maintaining the cycle of debt and reliance on gangs for her survival. In the case of 15 year old Parveen, her husband and in-laws suddenly left the slum after being involved in a gang dispute. She was pregnant at the time and had to deal with her pregnancy and delivery on her own. They later separated though Parveen hoped he would return despite his remarriage. Another two husbands were well-known troublemakers, whose wives enjoyed the status and power associated with their husbands. This in turn led to them being covertly disliked by some residents in their localities.

Some respondents had uncles or brothers who belonged to local gangs. Having a family member involved in these leadership alliances kept a person protected but also vulnerable to harassment, depending on the power politics at the time in the slums. Living in the city entailed the need for greater amounts of cash for day-to-day expenses. This required that the poor integrate into the labour economy. Lack of skills and education, however, were significant obstacles to integration with many remaining on the fringes holding temporary, poorly paid jobs. This in turn led many slum residents to depend on selling drugs as a way of making cash.

The presence of dealers, drug addicts and gang fights resulted in both adolescent and older women being wary of going out alone at night for fear of harassment and attacks by drunken men, gang members and others. For example, Morsheda, Jhorna and Mashida (married adolescent women) reported that at night they avoided going to the latrine located around the corner from their households, and used the space outside their homes for safety reasons. Such stories were typically mentioned in conversations and interviews. Married adolescent women also spoke of the fear of victimization within and outside their own compounds by landlords, and from male strangers in the slum and outside the locality. It was well known in section 4, that Harun, one of the landlords, regularly harassed his young female tenants by pinching their breasts, buttocks and brushing up against them when demanding rent. Some husbands also reported being careful about the kind of landlord they selected when they looked for a place to rent in the slums.

Arguments amongst leaders and rival gang members sometimes escalated into more dangerous violence, and there were incidents where gangs also sought revenge on rival member’s female friends and female family members. In one case reported, a young woman was gang-raped because she was accused of being an informer for a rival gang member (she was reportedly his girlfriend), and in another case, a leader’s sister was kidnapped by a gang member for one night as revenge, leading to stigmatisation of the young woman and her family in the community. Police were usually paid off and innocent men became victims of false police charges, in order to protect the real culprits. Slum leaders were both liked and hated by many of the residents. Sometimes, they were looked upon as patrons who provided support during crisis times, mediating in domestic disputes and amongst neighbours, and enforcing informal justice in the slums.

### Early marriage in slums: forced marriages versus love marriages

The direct and indirect costs of crime, gang feuds and violence can be rape, sexual harassment and early marriage, as families were keen to get their daughters married sooner in an environment of uncertainty and insecurity. The average age of marriage as reported was 13.5 years for 153 girls surveyed. Poverty and dowry (the illegal but widely accepted and practised payment of cash or goods to the groom) were very important motivating factors for many of the families, who married off their daughters early to avoid paying large amounts of money. Despite its illegality, dowry demands are still made, both before and after a marriage takes place. In the 153 interviews with married adolescent women, 70 of them disclosed that dowry ranging from Taka 5,000–31,000 (US $7–440) had been paid at the time of marriage. Dowry was referred to by most families and adolescent women as ‘demand’ with demands ranging from Taka 5,000 to 50,000 and above (with amounts increasing for wealthier families).

In the case of Mahfooza, the two key factors shaping her early marriage were an eruption of a cycle of gang violence from 2000-2001 and her father abandoning their household to remarry. Mahfooza was unhappy about being married off so young. She said, ‘*I did not want to get married*, *I was still in school but because of the bad situation in the slums you see I had to get married*, *because of the way the boys from neighbouring gangs were coming over and harassing girls...’*. The outbreak of violence and a change in the household income were therefore the main motivating factors for Mahfooza’s mother. She was running the household only with some support from her young son, after her husband had deserted her. Moreover, the climate of insecurity in the slum meant that Mahfooza’s mother was eager to see Mahfooza protected by her husband as the guardian, now that her father was gone.

Mahfooza was not the only young woman married off during this spate of violence in the slums; a number of others also mentioned poverty and gang threats as reasons for early marriage. A mother’s comments highlight the effects of on-going violence in the slums, which influenced her reasons for getting her 14 year-old daughter Parul married off quickly. She said, ‘*I had to protect my daughter…you see*, *this girl Sharmeen used to live in section 3*, *and then one night a few boys came over from another block and started shouting for her to come out of her room…she began screaming for help but none of us did anything*, *we were too scared…but thank Allah*, *they left soon after…it was that night that I made up my mind that I would marry her off*’.

In Bangladesh, in incidents of sexual harassment or rape, men are rarely held accountable and the victims often get blamed [12]. Similarly, it was observed in the slums that if young girls are harassed or even molested, there was little sympathy for their situation. In fact, parents and adolescent women spoke of the comments made by community members if girls were unmarried and seen to be “roaming around” the slums. An unmarried adolescent girl said, ‘*the people in this slum always have comments about me and my friends…why we are wandering around like this*, *don’t we have shame? I hear them telling my mother*, *aren’t you going to marry her off before she becomes too ripe?*’. Families feared scandals and loss of reputation or worse, sexual harassment, or rape, which would end any potential marriage prospects for their daughters.

Community norms and expectations and finding a ‘good husband’ also influenced when adolescent girls married. In a number of cases, marriages were hastily arranged by family members, particularly if they had found a ‘suitable boy’. Such hasty marriages meant that often families and even the adolescent women did not know enough about the groom or his family. With very little scope for discussions, this sometimes led to problems later in the marriage. In many cases, husbands or in-laws began to make financial demands after some months or years into the marriage, and commonly young women were beaten and harassed till the demand was met.

However, 81 of the 153 adolescent women reported marrying out of love and choosing their own partner. In the interviews, some young women repeatedly mentioned the phenomenon of love marriages in urban environments. Many of the young women were working in the garment factories in Dhaka city, which allowed them the opportunity to walk around freely and interact with males in the city environment [13]. Many of the girls spoke of what they saw as their right to choose their own partners, sometimes against their parents’ wishes, as they had an income and were independent. Others spoke of a new era where romance and love were the suitable ways to find partners. In this case, young women saw it as their right to choose their own spouses. As one young woman explained, ‘*why will my husband be selected by my parents…this is a new era and we can choose and marry who we want to…nowadays boys and girls select their own spouses’*. Young women spoke of changing times and expressed the view that it was ‘normal to fall in love in this day and age.’ Slums are very congested environments, and it was not uncommon to hear stories of young girls falling in love with boys who were tenants living in the same compound, or falling in love with brother’s friends and neighbours’ sons. Friends of young couples provided alibis and helped them find places to meet in private. One older resident in section 1 of the slum was known to rent out her room to drug users and young couples for a fee. Many of them spoke of going to parks and cinemas and walking around the city, or going on rickshaw rides (three wheeler bicycles) with their male friends.

Financial constraints also meant that some of the parents were unable to pay dowry or were reluctant to marry off their daughters since they (daughters) earned an income. In such cases, the girls may have been proactive in finding their own partners in the city environment. Most of the love marriages involved little or no dowry, but demands may be made at a later stage by the husbands themselves or the in-laws, which often led to marital instability. Twenty-six young women said their husbands and in-laws had demanded dowry payments after they had married.

### Regulating relationships: ensuring the reputations and honour of young women

Love affairs and relationships were regulated and monitored by community members, landlords and even gang members in the slum settlements. In a number of love marriages, community members, and family members intervened and quickly stepped in and forced a marriage if the young couples were found in a compromising situation, irrespective of whether the couple were ready to make such a commitment. In one case, a 15 year old girl was forcibly married off after being found in what was considered to be a compromising situation with her boyfriend. Three months after she became pregnant; he left her and she was alone, pregnant and without any security.

Leaders in the slum and many of the gang members played the role of moral guardians. There were reports of harassment, with young couples required to pay a fine if they were found spending time together alone. In one case, the boy was severely beaten and in another case, the young couple was forced to pay Taka 500 (US $7) as punishment to the gang members. In another incident in Phulbari slum, a group of boys confronted a young woman and a young man found together in a room, and accused them of flirting with one another. The young woman was forced to pay a fine of Taka 300 (US $4) and threatened with forced marriage if caught again. Many older community members justified the policing of young men and women’s behaviour. For them, the erosion of their authority combined with the perceived ‘flaunting of immoral behaviour’ of the younger generation left them distressed. A commonly heard remark made by some of the older residents was the ‘*lack of modesty and shame of young people in this era…*’. Many of the leaders and community members perceived their role as ensuring standards were maintained and actions taken to ensure that the ‘boy’ is forced to do the ‘right thing’ by the ‘girl’. A number of them said that forced marriage was critical for the adolescent girl. They did not necessarily view their role as enforcers but more as upholding the reputation of the female and her family. They argued that the young man is forced to be honourable and marry the young woman, ensuring her right to live in dignity in the community.

Sometimes, this type of ‘coercion’ was used as an opportunity by young women and gang leaders. As an unmarried adolescent girl, who was close to one of the gang members, explained, ‘*the* (*gang leader*) *said to me*, *“why don’t you bring your boyfriend over and spend some time in the room and we will come into the room and force him to marry you and claim he compromised your reputation”…leaders do this if the girls pay them Taka 500-1000*’. The adolescent girl did not take the leader up on his offer. It is unclear how common this practice is. In some cases, leaders are found to be involved in extra-marital affairs or harassing young women themselves, but this is generally overlooked in the community.

### Marital instability

Narratives among married adolescent women reveal that increasingly there is less security and certainty tied to marriages in the slums, particularly for love marriages, which are usually arranged without dowry and often without the guardians’ permission. In addition, poverty, unemployment, drug use, and lack of acceptance from in-laws were all mentioned as contributing factors for marital instability. Of the 153 adolescent married women interviewed, 24 said they were separated from or abandoned by their husbands, another seven young women revealed this was their second marriage, and with further probing, seven young women were found to be sharing their husbands with a co-wife. The law in Bangladesh does not ban polygamy but it is meant to be restricted and controlled. A man wishing to marry again needs the written permission of his first wife, but in reality the woman’s permission is rarely sought and related laws are totally ignored. It was difficult to get young women to share their true marital status, as much social disapproval and stigma surrounds divorce in Bangladesh. Most of the female adolescents were aware of the stigma of divorce and separation, and desertion was viewed as a personal failure on their part.

Adolescent women said that separated young women were often gossiped about, insulted during quarrels and referred to as ‘*bad women*’, and as ‘*a wife who could not eat her husband’s rice*’. The comment was aimed at women to imply they were intolerant and difficult wives who were unable to maintain the harmony in a marriage. *‘Not being able to eat a husband’s rice’* is a serious insult as it casts aspersions on a woman’s character and personality.

Stories were shared of how adolescent women found out about ‘other women’ or co-wives, because of their husbands’ suspicious behaviour (spending time away from home, rumours of remarriage by neighbours, less income given to the wife, etc), but rarely did any of the men confess to their wives. Some left their wives, others continued to live in two households, going back and forth between slums. Many women were reluctant to admit their husbands had married again, and a common refrain was, *‘everyone will say she couldn’t eat her husband’s rice… [she is difficult]…that is why he left her and now she has no home*, *no support…’*.

In discussions with married adolescent women, it was apparent that many of them experienced anxiety about abandonment and separation. In some cases, the marriage was never officially ended. Either the husband deserted the woman or she left because of severe abuse, and they both remarried, or in some cases the husband reappeared and the wife accepted him back. In Majeda’s case, her husband suddenly left her after three years of marriage. She waited for a year for him to return and after that she married another young man she met in the slum. In Rehana’s case, she fell in love and married her co-worker and fell pregnant soon after. After a few months, her husband suddenly disappeared, leaving her pregnant and vulnerable. She ended up terminating her pregnancy. In the case of Lipi, her husband left her after she could not pay the dowry money demanded by his family. It was rumoured that he had remarried, and Lipi had become a sex worker to support herself and her family.

Alongside the insecurity and inability to track errant husbands who may leave suddenly and move to another city or slum, slums can afford certain kinds of freedom. Young women spoke of the ability for women to remarry, relocate and also start their lives and relationships anew as living in a city afforded a certain kind of mobility and privacy. In the case of Rashida, after her first marriage failed, she began a relationship with a married migrant in the slum. Soon after eviction, she moved to another slum and continued her relationship without any fear of reprisals as no one knew her or his marital history. There were also stories of young women who had allegedly been unfaithful to their husbands and vice versa. These kinds of indiscretions may be tolerated or result in fights and then neighbours or kin mediate to bring spouses back together. While as noted there are social sanctions, women also spoke of the forced interference from family and neighbours in villages. In contrast, in certain slums, particularly very large ones, households were more fragmented and less tied down by kinship and family networks. Thus, there was less chance of interference as many households remained anonymous.

### Violence and insecurity in the household

Many adolescent women said that food scarcity in the households often led to arguments about unpaid dowry payments and that physical violence ensued. Of the 153 adolescent women surveyed, 89 said they had experienced regular to occasional violence from their husbands. Forty-four women admitted that in the past six months they did not have enough money to buy food and borrowed on credit from shops. This led to uncertainty, arguments and fights in the household. For example, Sohely’s husband faced unemployment and life was difficult at home. She said, ‘*We owe rent and there is no food for the past few days in the house. I told him to go to work. Just because I told him to go to work he turned on me and started screaming*, *“Beggar’s daughter you brought no* (*dowry*) *money and then you tell me to work. And where will I go to work…”. He started slapping me and pushed me off the bed…’*.

In the case study interviews, when I had informal discussions with a few of the husbands, they admitted to the violence but justified their behaviour by stating that they felt ‘*useless as men when they were unable to provide for their families’*, and remained *‘anxious about their inability to find permanent jobs’*. A few of them also spoke of the reversal of roles in the city, where ‘*women are no longer in the homes but working in factories’* while men were unable to manage their households. Some of the adolescent women, on the other hand, reported that their husbands were *‘lazy’* and *‘did not like to work’*, preferring to *‘gamble*, *drink alcohol and take drugs’*. Twelve married young adolescent women reported that their husbands were drug addicts. Usually during incidents of violence, it was observed that community members did not get involved as it was seen as a ‘private family matter.’ According to Roshonara (adolescent woman), if there were any fights with one’s husband, usually the neighbours sided with the men. She said that when her husband hit her, no one said anything, but when she fought back no one comforted her: ‘*He does not like to work but our society attacks the women but not men. If I say a few words to him everyone remembers every word I said. They say*, *“Oh Roshonara is so bad*, *look at the way she behaves with her husband”’*.

### Childbearing: ‘the woman who cannot have a child has no strength’

Of the 153 married adolescent women, 128 had children soon after marriage. Fears of marital insecurity and the need to tighten marriage bonds and hold on to their husbands were alluded to by a number of adolescent women. Married adolescent women complained that with poor employment prospects in the city and unhappy about unpaid dowry, men easily deserted their wives, relocated and remarried economically solvent women. Other men were lost in a cycle of substance abuse. But married adolescent women spoke about how they had seen that in many households, children had made ‘husbands more responsible’ and ‘strengthened marital bonds’, and many claimed this was the case for them. Bearing a child was seen as important, particularly if it was a love marriage, and with no dowry paid, it was hoped the birth of the child would bridge any tensions with in-laws and bring more support from other family members in the household.

Narratives revealed an increase in confidence and security after the birth of a child. As one adolescent woman explained, '*After the birth of my son my strength increased. Also once you have a child*, *a son then there is proman* (*evidence*)*. If anything happens you can get a hold of the man. The woman who cannot have a child has no strength. When my mother-in-law and sister-in-law would treat me really badly*, *my neighbours would say to my husband*, *‘you have a son now. She has given birth to a child so why do you let them give her so much of a hard time?'.* Other comments were *'my mother-in-law loves my baby and is much nicer to me since the birth of my child*', and *'he* (*husband*) *now gives me his income whereas before he would give his sisters and parents his income*'. While some of the adolescent women claimed that they wanted to wait for some time before having children, they were in a dilemma, torn between competing demands of in-laws, husbands, their circumstances and strong cultural pressures to prove one's fertility soon after marriage. But in informal discussions both older and younger women admitted that a child did not necessarily ensure a secure marriage or that the husband would stay faithful. An adolescent woman with three children said, *'If we don’t have children it is a problem – men want to go and marry someone else. But then again also if you have children men go off and you are left to look after the children*, *so you don’t really win either way...'*.

### Pregnancy and terminations: difficult choices, risky experiences

Out of 153 married adolescent women, 27 reported that they had terminated a pregnancy. Of these, 13 had sought legal services and 14 had illegally aborted the pregnancy, relying on unsafe methods. While many married adolescent women in the slum value children, they are not keen to have large families because of the poverty and suffering in their lives. Some of the young women spoke of the difficulties of feeding and looking after more than one child. Many of the young women have incorporated the discourse of family planning campaigns, which link smaller family size to better economic conditions, and hope to improve their quality of life. Only a few women revealed moral or religious concerns with termination. Some of the working women preferred to keep it quiet from others because they worried that neighbours would assume the abortion was related to an extra-marital affair. As one young woman garment factory worker explained, *‘They say to women*, *why don’t you want to keep the baby? Is it not your husband’s?’*.

Eleven married adolescent women said they felt compelled to terminate pregnancies against their wishes due to circumstances in their households, from in-laws not accepting the marriage, presence of a second wife, financial powerlessness, desertion, and inability to fight with family members to keep the baby. The process of getting a termination can be risky, as young women often opted for illegal services, or the delay in decision-making meant that the pregnancy termination was risky. One case below exemplifies the situation for many poor young women:

Fatima did not want to terminate her second pregnancy, however her husband insisted they could not afford another child, and he also had a second wife to support. Fatima admitted that her marriage was volatile but wanted another baby to ‘hold on to her husband’ and believed his affections would wane towards the other wife if she could have a son. By the time Fatima realised that her husband would not support her, she was already more than four months pregnant, and she ended up seeking treatment from a local pharmacy and purchased some ‘pills’. When that did not work, she visited a homeopathic doctor. She developed post-abortion complications and had to be rushed to hospital.

### Protecting oneself: putting up or facing more stigma and discrimination

The slum environment combined with patriarchal structures, social and cultural pressures and the lack of economic power, forced many adolescent women to tolerate their husband’s behaviour for their own social and economic protection, and to avoid the stigma of being single. When asked why they didn’t just leave their difficult husbands, common statements made among the adolescent women were, ‘*Is it so easy to leave the husband? Can I just leave him?’; ‘How many times will a girl get married in her life? What if the second husband is worse than this one?’*. Married adolescent women spoke of a biased society that did not support women; rather it was biased towards men. They were pragmatic and realised that if they left their husbands, as poor single women they would face possible assault, discrimination, and experience both social and economic insecurity. A young woman stated, *‘it is better that I tolerate my husband’s bad behaviour*, *if I leave him*, *I know that I will be noticed and harassed by the other [bad] men and others in the slum…I am married now with a child*, *how will I manage if I leave him now!’*. Lack of a male protector left adolescent women feeling extremely vulnerable to exploitation and threats of sexual harassment, particularly if there was no male family or kin nearby, willing and able to provide protection and support.

In the absence of financial autonomy, some adolescent women (widowed, abandoned or separated) relied on their youth and attractiveness to get males to court them, in some cases, taking them on as lovers in exchange for financial support, irrespective of the man’s reputation. Males with power and networks offered more than just financial support but also ensured protection and status in the community. Observations and informal discussions found that some widowed or divorced women took on lovers to ensure their well-being and rights to access privileges (water, gas, electricity, schools for their children, jobs). Thus, men, particularly if well placed and employed, were seen as a source of security and protection. Single women and women without any family support or income are particularly vulnerable to sexual harassment. For those poor adolescent women who work outside the home, the employment market is structured against them, with less scope to move up and develop their skills. As Jesmin and Salway note, there are some opportunities for adolescent women in garment factories and domestic labour; but compared to men, scope for upward mobility in labour market and job options is far more constrained [14].

Through the narratives and lived experiences of married adolescent women, it becomes apparent that the individual politics of reproductive and sexual behaviour and meanings of ‘rights’ are embedded in larger socio-cultural, political and economic inequalities, and in the familial relationships in their lives. There are various levels of ‘structural violence’ operating on the lives of urban poor adolescent women and their families, impacting on the way rights are understood and exercised. As my research found, a large number of married adolescent women marry as early as 14 to 15 years because of crime and insecurity. However, many also elope with lovers and marry early, and perceive their right to choose a partner (love marriage) as a sign of changing times in the city. A few of the married adolescent women also spoke of ‘their right’ not to have forced marriages and many argue that they are entitled to select their own spouse. The reasons are often complex as discussed. Here we have two different perceived entitlements of rights, both of which impact on sexual and reproductive health decisions for young women and have consequences for them and their families. They also affect young adolescent women’s capacity to claim entitlements.

What does ‘rights’ mean in a setting where lives are highly constrained? If a girl aged 15 decides to have a child for the reasons described, then can we say she is exercising her reproductive rights? If she is married off below the legal age to avoid the sexual predations of other men, what does this tell us about the disconnection between the formal environment of legal rights and the realities of these young women’s lives? The point is that young women and their families remain pragmatic and often do not have the opportunity to exercise their rights as citizens. In the case of reproductive health decisions about pregnancy and health promotion messages where married adolescent women are asked to delay child birth by health providers, this message is not always amenable to the reality of their lives. Many choose to have children early, for emotional, social and strategic reasons, citing ‘bonding in marriage’, ‘making in-laws happy’, and closer relationships with family as a result of their decisions. Some terminate their pregnancies and often blame factors outside their control for their decisions, from economic constraints to pressures from husband and in-laws etc. Another key point is the disconnect between young women’s sense of their entitlements or rights versus a universal discourse of rights. If a 13 or 14 year-old willingly marries and has sex with her husband because she feels it is her right to marry for love, what kind of rights is she exercising? Similarly, many married adolescent women bear children early and it is seen as a natural progression soon after marriage. This is reinforced by the community, and particularly self-appointed local leaders who decide on social sanctions, from fines to public humiliation, including forced marriages to ensure young men’s and women’s relationships and behaviour are regulated and reputations remain intact.

## Conclusions

ICPD is the first United Nations population policy document that endorses a range of rights applicable to women's reproductive health and security, and recommends that national population policies respect international human rights norms [1]. Of the international human rights instruments to which the Government of Bangladesh is party, the Universal Declaration of Human Rights and the Convention on the Elimination of All Forms of Discrimination Against Women (Women's Convention) are preeminent in protecting reproductive rights [15]. Reproductive health issues are addressed through a variety of laws and policies in the country. The manner in which these issues are addressed is seen as reflective of a government’s commitment to advancing reproductive health and rights of women in the country. Bangladesh has good examples of laws relevant to women’s reproductive health rights. These include a minimum age of marriage of 18 years, and the legal proscription of the practice of dowry. There are women-focused policies using incentives to encourage education for young girls and services on family planning and maternal health, all of which are in place to empower women. The reality is that there is a gap between policies, their implementation and the lived experiences of poor adolescent women and their families living in slums in the country, who remain socially excluded.

Carla Obermeyer argues that less attention has been devoted to the ICPD conceptual framework that guides the formulations of objectives particularly when one applies the notion of reproductive rights from a global perspective to diverse local settings, where cultural and economic perspectives shape understandings of reproductive health. She argues for a reflexive stance and recognition that those who are the target of policies and those who formulate and implement policies –are in an unequal position [16]. As this paper outlines, the adolescent women and the communities which reside in slums may not necessarily perceive the rights promoted as more rational or more desirable, given the social, cultural, political, and economic condition of their lives. Many of the adolescent women and their families may perceive the laws and messages of delayed marriage, birth spacing, and delayed childbearing with a degree of ambivalence. If one were to tease apart and examine lived experiences in the slums for adolescent women and the community, one is confronted with the many different, often contradictory meanings and negotiation of *rights* among the young women and the community. Some practices generally thought to impact adversely on adolescent women’s position from the universal perspective of rights, were seen as empowering. For example, most adolescent women’s willingness to take on practices (e.g. early marriage, bearing children) which can be seen as adversely affecting their health and well-being demonstrates an acceptance of the cultural environment and norms in which they reside, but at the same time a practical and strategic understanding of their limited options. These practices may gain a greater degree of support from husbands and in-laws, or even independence or the achievement of better socioeconomic conditions for themselves and their families. The community leaders’ enforcement of marriages among the adolescent women and their ‘suitors’ is viewed as ‘a right of the leaders’ to ensure order, rather than perceived as a violation of human rights. In this environment, communities, families and especially married adolescent women in Dhaka slums understanding of their rights, in terms of the way they negotiate their everyday lived experiences and make sexual and reproductive health decisions and ‘choices’, is very different from the widely accepted discourse on rights globally, which assumes a particular kind of individual thinking and discourse on rights and a certain autonomy women have over their bodies and their lives. This does not necessarily exist in urban slum populations. Notions of rights and entitlements are far more complex in environments where women and men live in conditions of poverty and socioeconomic deprivation. Unless these notions and their link to underlying conditions are also addressed, sexual and reproductive health rights will remain an unreal prospect for many young women.

## List of abbreviations used

AL: Awami League; BNP: Bangladesh National Party; ICPD: International Conference on Population Development; NGOs: Non-Governmental Organizations; SRH: Sexual and Reproductive Health; SRHR: Sexual and Reproductive Health and Rights.

## Competing interests

No competing interests declared.
